# Rapunzel Syndrome: Diagnostic Challenges and Multidisciplinary Treatment Strategies

**DOI:** 10.7759/cureus.61294

**Published:** 2024-05-29

**Authors:** Mihir Patil, Pankaj Gharde, Raju K Shinde, Pratik S Navandhar

**Affiliations:** 1 General Surgery, Jawaharlal Nehru Medical College, Datta Meghe Institute of Higher Education and Research, Wardha, IND

**Keywords:** imaging investigations, halitosis, gastrointestinal complications, endoscopic examination, decomposing hair, colon, cognitive-behavioural therapy, behavioural therapy, anaemia, abdominal pain

## Abstract

Rapunzel syndrome, a rare yet complex condition, poses diagnostic and therapeutic challenges. Trichobezoars, stemming from trichotillomania and pica, manifest as hair conglomerates within the gastrointestinal tract, often necessitating surgical intervention. This review synthesizes literature on symptomatology, diagnostic methods, and treatment modalities, emphasizing the multidisciplinary approach essential for effective management. Psychological interventions, including cognitive-behavioral therapy, complement surgical measures in addressing underlying psychiatric factors. Diagnostic imaging, endoscopic examinations, and histopathological analysis aid in an accurate diagnosis. Enhanced awareness among healthcare providers regarding the association between psychological disorders and gastrointestinal complications is crucial for timely intervention and improved outcomes in individuals with Rapunzel syndrome.

## Introduction and background

A very uncommon digestive disorder in people caused by eating hair is called Rapunzel syndrome (trichophagia) [1]. Trichophagia is occasionally linked to trichotillomania, which causes excessive hair-pulling [2]. The long-haired princess Rapunzel from the Brothers Grimm fairy tale inspires the name Rapunzel syndrome [3]. Trichobezoars are an uncommon medical ailment that frequently necessitates surgery and is frequently linked to an underlying mental illness [4]. They usually reside in the stomach, but as they become more significant over time, they may spread like a tail through the pylorus into the distal portions of the small intestine [5,6]. Any adolescent patient with psychiatric illnesses, intestinal occlusion or sub-occlusion symptoms, or a history of trichotillomania should have the option of Rapunzel syndrome taken into consideration [7]. Patients frequently arrive with symptoms of blockage, nausea, vomiting, and abdominal pain [8]. The colon, ileum, or jejunum could contain the distal end of the bezoar [5,8]. It typically has nebulous symptoms when it first appears, but complications like perforation, peritonitis, and obstructive jaundice can also occur [9]. The exact prevalence and incidence of Rapunzel syndrome are not well documented due to its rarity. However, trichophagia is estimated to occur in about 10-30% of individuals with trichotillomania, which has a prevalence of about 1-2% in the general population [2,4]. Rapunzel syndrome is even less common, with fewer than 100 cases reported in the medical literature [1,6].

## Review

Search methodology

**Figure 1:**
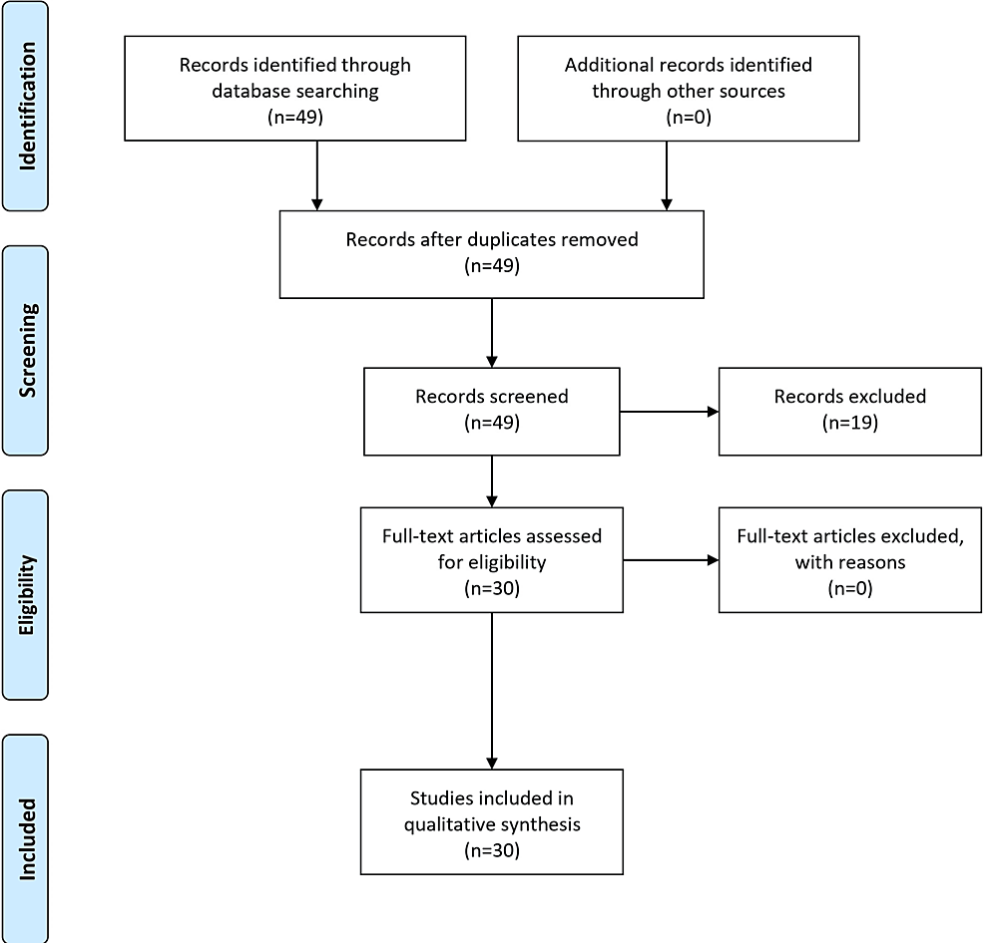
PRISMA flow chart PRISMA: Preferred Reporting Items for Systematic Reviews and Meta-Analyses Created by Mihir Patil

The search methodology followed Preferred Reporting Items for Systematic Reviews and Meta-Analyses (PRISMA) guidelines, utilizing PubMed/MEDLINE, Google Scholar, and medical journals. Keywords and Medical Subject Headings (MeSH) terms related to Rapunzel syndrome, trichobezoar, trichotillomania, and psychological interventions were employed. No language or date restrictions were applied. The inclusion criteria targeted studies on symptoms, diagnosis, psychological factors, and treatment approaches for Rapunzel syndrome, excluding unrelated conditions. Manual screening of reference lists supplemented with electronic searches. Data extraction and synthesis were independently conducted, with discrepancies resolved through consensus. Finally, selected studies were critically appraised for relevance and quality, ensuring a comprehensive and systematic review process. The PRISMA flow chart is shown in Figure 1.

Symptoms and complications of Rapunzel syndrome

Trichobezoars are hair-based foreign bodies typically found in the stomachs of children and adolescents with trichotillomania [2,5]. However, they can also extend through the pylorus into the distal portions of the small intestine, where they form a structure resembling a tail and are referred to as Rapunzel syndrome [10]. There are no specific diagnostic criteria for Rapunzel syndrome in the International Classification of Diseases (ICS) or the Diagnostic and Statistical Manual of Mental Disorders (DSM). However, related conditions like trichotillomania and trichophagia are recognized in the DSM-5 [1,11]. The size of the hairball and its location within the digestive tract are two variables that can affect the symptoms of Rapunzel syndrome [11]. People may initially feel pain in their abdomens, ranging from a slight ache to intense cramping [4,10,12]. Feelings of fullness or bloating may accompany this discomfort since the hairball occupies stomach space and impedes proper digestion [13]. Rapunzel syndrome frequently manifests as nausea and vomiting, which are the body's attempts to get rid of the indigestible hair [14]. Hair strands found in vomit might be concerning and suggest an underlying problem [6,15]. People may also experience a chronic loss of appetite, which, over time, may result in unintentional weight loss [16]. In more serious situations, the hairball may result in intestinal blockage, a potentially fatal consequence [8]. This happens when the hairball partially or obstructs the intestines' ability to pass waste and food [9]. Depending on the location and degree of the blockage, symptoms of intestinal obstruction can include severe stomach discomfort, constipation, or diarrhea [4,17]. Intestinal blockage can cause major side effects such as tissue damage, infection, and even colon perforation if left untreated [17]. Decomposing hair lodged in the digestive tract can also cause halitosis, or bad-smelling breath, another sign of Rapunzel syndrome [18]. Rapunzel syndrome symptoms are described in Table 1.

**Table 1:** Rapunzel syndrome symptoms

Symptom	Description
Abdominal pain	Ranges from mild aches to severe cramping
Bloating/Fullness	Feels full due to hairball occupying space in the stomach
Nausea and vomiting	The body's attempt to expel hair
Hair in vomit	This may indicate Rapunzel syndrome
Loss of appetite	Can lead to weight loss
Intestinal blockage (serious)	Potentially life-threatening
Blockage symptoms	Severe abdominal pain, constipation, or diarrhea
Long-term blockage complications (serious)	Tissue damage, infection, perforation
Bad breath (halitosis)	Decomposing hair in the digestive tract

Diagnostic methods for Rapunzel syndrome

Diagnostic imaging investigations, such as abdominal ultrasound, computed tomography (CT) scans, or magnetic resonance imaging (MRI), are commonly used in the diagnosis of Rapunzel syndrome to visualize the existence and extent of the trichobezoar [7,19,20]. However, while treating youngsters, the possibility of radiation-induced cancer is a crucial factor to take into account [21]. Additionally, endoscopic examination may be used for direct visualization and biopsy to diagnose this condition properly [22,23]. Anemia or electrolyte abnormalities caused by malnutrition or intestinal blockage may be discovered through laboratory testing [20]. Gastroscopy can be performed, and a tumor sample can be taken for histopathological examination, which confirms the trichobezoar diagnosis [23]. The diagnostic techniques for Rapunzel syndrome are described in Table 2.

**Table 2:** Diagnostic techniques for Rapunzel syndrome Created by Mihir Patil

Diagnostic technique	Description	Advantages	Disadvantages	Considerations
Abdominal ultrasound	Uses sound waves to create images of the abdomen	Safe, readily available, relatively inexpensive - Good for initial evaluation of suspected trichobezoar	Limited visualization of soft tissues	Preferred for initial investigation, especially for children
Computed tomography (CT scan)	X-ray imaging technique to create detailed cross-sectional images of the body	Provides detailed images of the abdomen and pelvis - Can precisely determine the size, location, and extent of trichobezoar	Involves ionizing radiation, which carries a small cancer risk	Use with caution, especially in young patients. Consider alternatives like ultrasound first
Magnetic resonance imaging (MRI)	Uses strong magnetic fields and radio waves to produce detailed images of organs and soft tissues	No ionizing radiation - Excellent for visualizing soft tissues	Expensive and may not be readily available in all healthcare settings	Consider if ultrasound or CT scan is inconclusive or if more detailed soft tissue imaging needed
Endoscopy (upper GI endoscopy)	Uses a thin, flexible tube with a camera to directly visualize the upper gastrointestinal tract	Allows direct visualization and potential biopsy of the trichobezoar	Can be uncomfortable for the patient	May be used to confirm diagnosis and assess severity
Laboratory tests	Analysis of blood or other bodily fluids	May reveal anemia, electrolyte imbalances indicative of malnutrition or blockage	Indirect diagnostic method	Can provide supportive evidence for Rapunzel syndrome

Psychological factors contributing to Rapunzel syndrome

Rapunzel syndrome [4] is caused by hair ingestion. It is a compulsive behavior in which sufferers ingest their own hair in addition to pulling it out. The primary psychiatric disease known as trichotillomania is typified by an overwhelming impulse to pull out one's own hair, resulting in substantial hair loss [24]. This obsessive behavior can be upsetting and challenging to stop, which frequently causes a major impairment in day-to-day functioning [4,24]. Another psychological condition that is frequently connected to Rapunzel syndrome is pica [10]. The Latin word for "magpie," which is infamous for its indiscriminate eating habits, is where the name "pica" comes from [25]. Pica, as it relates to mental health, is the incessant desire and ingestion of non-food materials like wool, hair, linen, or even tiny metallic objects [7,25]. Depending on the substance consumed, this condition may pose serious health hazards, such as gastrointestinal blockage or poisoning [15].

Treatment approaches for Rapunzel syndrome

Surgical Treatment Options for Rapunzel Syndrome

The treatment for Rapunzel syndrome is interdisciplinary. An endoscope, a flexible tube with a camera and equipment attached, may remove a small trichobezoar if it hasn't caused severe blockage [26]. Larger trichobezoars or those causing complications like intestinal obstruction may require surgical intervention. These larger masses or those producing problems frequently need to be surgically removed using a laparoscopy (minimally invasive surgery) or laparotomy (open surgery) [5,27]. Laparoscopic surgery, or minimally invasive surgery, involves making tiny abdominal incisions through which various surgical equipment and a laparoscope, a thin, flexible tube with a camera, are introduced [27,28]. Then, without making a huge incision, the surgeon makes his way through the abdominal cavity to find and remove the bezoar [29]. Compared to laparotomy, i.e., open surgery, laparoscopic surgery has several benefits, such as shorter recovery periods, less pain after surgery, and smaller scars [23,29]. Nevertheless, the patient's general health and the size and position of the bezoar will determine whether it is appropriate [14]. Table 3 illustrates the surgical treatment for Rapunzel syndrome.

**Table 3:** Surgical treatment for Rapunzel syndrome

Surgical treatment option	Description	Advantages	Disadvantages	Suitability
Endoscopy	Flexible tube with camera used to remove small trichobezoars	Non-invasive	Limited to small bezoars, may not be effective for complete removal	Small, uncomplicated trichobezoars
Laparoscopy (minimally invasive surgery)	Tiny abdominal incisions for instruments and camera to remove bezoar	Shorter recovery time - Less pain - Smaller scars	More technically challenging - May not be suitable for very large bezoars	Bezoars of moderate size, good patient health
Laparotomy (Open Surgery)	Large abdominal incision to access and remove bezoar	Effective for large or complex bezoars	Longer recovery time - More pain - Larger scar	Large bezoars, complications present

Psychological and behavioral interventions for Rapunzel syndrome

Trichophagia (a hair-eating problem) and trichotillomania (a hair-pulling disorder) are two underlying psychological conditions that are frequently linked to Rapunzel syndrome [23,24]. Consequently, psychological assessment and assistance are crucial to treat the underlying reason and stop recurrence [28]. In extreme situations where the trichobezoar has brought on malnourishment or other nutritional deficiencies, nutritional supplementation could be required to restore health [22,29]. People with Rapunzel syndrome may benefit from behavioral therapy and cognitive-behavioral therapy (CBT), which may assist them in overcoming the obsessive tendencies connected to the condition [30]. CBT, a primary intervention, helps patients recognize and change the thought patterns and behaviors contributing to hair-pulling and ingestion [21]. Habit reversal training (HRT), a specific type of CBT, is also effective in managing trichotillomania by teaching patients to substitute hair-pulling with less harmful behaviors [23]. In addition to CBT, other therapeutic approaches like dialectical behavior therapy (DBT) and acceptance and commitment therapy (ACT) have shown promise [8,20]. These therapies focus on emotional regulation and acceptance strategies, which can be beneficial for individuals with co-occurring mood disorders [7,29]. Continuous monitoring and psychological care are essential to address any underlying mental health concerns and prevent recurrence even after the trichobezoar has been successfully removed [6]. Effective psychological management is crucial given the strong association between trichophagia and psychiatric conditions such as trichotillomania, obsessive-compulsive disorder (OCD), and anxiety disorders [1,28].

Pharmacological treatment can complement psychological therapies, particularly in cases where comorbid psychiatric disorders are present [2]. Selective serotonin reuptake inhibitors (SSRIs) such as fluoxetine and sertraline are commonly prescribed to manage symptoms of anxiety, depression, and OCD, which often co-occur with trichotillomania and trichophagia [4,23]. Antipsychotic medications like olanzapine have also been used, particularly for their sedative properties and ability to reduce obsessive-compulsive behaviors [7,25]. N-acetylcysteine (NAC), an over-the-counter supplement, has shown efficacy in reducing hair-pulling behaviors in trichotillomania due to its role in glutamate modulation [13,17]. While the evidence for NAC, specifically in trichophagia, is limited, its success in related conditions suggests potential benefits [25].

Despite the progress in understanding and managing Rapunzel syndrome, several gaps remain in the literature. Most notably, there is a lack of large-scale, controlled studies that evaluate the long-term efficacy of combined psychological and pharmacological treatments [11,14]. Additionally, the specific mechanisms by which trichophagia leads to trichobezoar formation and the most effective strategies for preventing recurrence post-surgery are not well documented [11,14,23]. Longitudinal studies examining the outcomes of different therapeutic interventions would also provide valuable insights into the most effective treatment combinations [23]. Further research is needed to explore the genetic and neurobiological factors predisposing individuals to trichophagia and trichotillomania.

## Conclusions

Rapunzel syndrome, a rare condition characterized by trichobezoars in the gastrointestinal tract due to trichotillomania and trichophagia, presents significant diagnostic and therapeutic challenges. Symptoms include abdominal pain, bloating, nausea, vomiting, and severe complications like intestinal blockage. Effective diagnosis involves imaging techniques such as ultrasound, CT scans, MRI, and endoscopic examinations. Treatment often requires surgical removal of the trichobezoar, with options ranging from endoscopy for smaller masses to laparoscopic or open surgery for larger ones.

Psychological interventions are crucial due to the syndrome's strong association with psychiatric disorders. Cognitive-behavioral therapy (CBT), particularly habit reversal training (HRT), and pharmacological treatments like SSRIs and antipsychotics are effective in managing underlying compulsive behaviors. Despite progress, gaps remain in understanding the long-term efficacy of combined treatments and the mechanisms leading to trichobezoar formation. Future research should focus on large-scale studies and explore genetic and neurobiological predispositions. Enhanced awareness among healthcare providers about the connection between psychological disorders and gastrointestinal complications is essential. A multidisciplinary approach, integrating surgical and psychological care, is vital for effectively managing Rapunzel syndrome and improving patient outcomes.
